# The Influence of Centre-Based Childcare on Preschoolers’ Physical Activity Levels: A Cross-Sectional Study

**DOI:** 10.3390/ijerph110201794

**Published:** 2014-02-05

**Authors:** Leigh M. Vanderloo, Patricia Tucker, Andrew M. Johnson, Melissa M. van Zandvoort, Shauna M. Burke, Jennifer D. Irwin

**Affiliations:** 1 Health and Rehabilitation Sciences, Faculty of Health Sciences, University of Western Ontario, London, ON N6G 1H1, Canada; 2 School of Occupational Therapy, Faculty of Health Sciences, University of Western Ontario, London, ON N6G 1H1, Canada; E-Mail: ttucker2@uwo.ca; 3 School of Health Studies, Faculty of Health Sciences, University of Western Ontario, London, ON N6A 5B9, Canada; E-Mails: ajohnson@uwo.ca (A.M.J.); sburke9@uwo.ca (S.M.B.); jenirwin@uwo.ca (J.D.I.); 4 Central East Tobacco Control Area Network (TCAN), Simcoe Muskoka District Health Unit, Barrie, ON L4M 6K9, Canada; E-Mail: melissa.vanzandvoort@smdhu.org

**Keywords:** preschool-aged children, accelerometry, childcare environment, health promotion

## Abstract

The childcare environment represents an appropriate avenue to support physical activity among preschoolers. The aim of this study was two-fold: (1) to measure the physical activity levels of a sample of preschoolers during childcare hours; and (2) to assess which attributes (e.g., space, equipment, policies) within centre-based childcare environments influenced physical activity. Thirty-one preschoolers from 5 childcare centres across London, Canada participated. Actical accelerometers were worn by participants for one day during childcare hours to assess activity levels using a 15 second epoch length. The Environment and Policy Assessment and Observation instrument was used to conduct a full-day evaluation of the childcare environment. On average, participants engaged in 1.54 min/h of moderate-to-vigorous physical activity and 17.42 min/h of total physical activity. Sedentary opportunities, portable and fixed play equipment, and staff behaviour accounted for 49.3% of the variability in moderate-to-vigorous physical activity and 14.1% of the variability in total physical activity, with sedentary opportunities, fixed play equipment, and staff behaviours displaying an inverse relationship. Results emphasize the critical role the childcare environment plays in supporting physical activity among preschoolers.

## 1. Introduction

Physical activity is central to the health and wellbeing of young children [1]. Unfortunately, research suggests that preschoolers are insufficiently active [2]. In fact, Tucker’s [2] review found that only 54% of studies reported that preschoolers participated in a 60 min of physical activity per day. This finding is sobering given the many associated health benefits to physical activity among this young cohort; including the promotion of healthy weight gain, cardiovascular health, and positive cognitive development [1]. Not surprisingly, the importance of intervening during the preschool years is supported by the fact that physical activity behaviours established in early childhood continue through one’s lifetime [3]. Ignoring the significance of preschool physical activity will undoubtedly prove detrimental in terms of health and development.

The large proportion of preschoolers in childcare (~80%) [4], coupled with the number of hours children spend in this environment (~29 h/week) [5], support this venue as ideal for promoting physical activity among this population. International research suggests that this environment has a strong influence on the physical activity levels of preschoolers, accounting for approximately 50% of the variability [6]. Similarly, the findings of a recent meta-analysis suggest that the childcare environment is an effective venue to support moderate-to-vigorous physical activity (MVPA) among preschoolers [7].

Few studies have explored the relationship between childcare centres and preschoolers’ physical activity [6,8,9]; specific characteristics within this environment have been associated with higher levels of physical activity, including: gross motor equipment (*i.e.*, portable play equipment like balls, tricycles which support large muscle movement); sufficient indoor/outdoor play space; and childcare providers’ level of training. However, despite this particular setting being acknowledged as significant with regard to preschoolers’ activity levels, Canadian studies exploring the associations between preschoolers’ physical activity in centre-based childcare have not yet been conducted. Given that long periods of sedentary behaviours are common in childcare [6,10], coupled with the increased risk for obesity among Canadian children who attend centre-based childcare [11], greater attention is required to address the low levels of physical activity among Canadian preschoolers. Moreover, greater insight is required into the environmental attributes of childcare facilities that best support and maintain this health promoting behaviour.

Limited Canadian research has objectively assessed physical activity among this population [1,12]. In fact, no Canadian research exists measuring the activity levels of preschoolers during centre-based childcare hours, or examining the influence of this environment on preschoolers’ physical activity. Therefore, this is the first Canadian study aimed at measuring objectively the physical activity levels of preschoolers in childcare as well as assessing which attributes (e.g., equipment, staff behaviour) within the centre-based childcare environment influenced physical activity.

## 2. Methods

### 2.1. Participants and Study Design

A cross-sectional study was undertaken; 13 publicly-funded childcare centres from a large organization in London, Ontario were invited to participate—of these, five agreed. A total of 31 preschoolers (mean age = 4.10 years, *SD* = 0.85; 17 male; response rate = 60%), across the five childcare centres (from the same organization), received parent/guardian consent to participate.

### 2.2. Data Collection

Prior to data collection, parents/guardians were asked to complete a demographic questionnaire. Activity data were collected by Actical accelerometers (MiniMitter, Bend, OR, USA), using a 15 sec epoch length. Having demonstrated acceptable reliability and validity in measuring preschoolers’ physical activity [13], these omnidirectional devices provided detailed information on movement counts and activity intensities. Participants were fitted with a device for 1 day during childcare hours. Accelerometers were securely fastened to the children’s right hip at the start of the day and were removed prior to them leaving the centre.

The physical activity component of the Environment and Policy Assessment and Observation (EPAO) instrument was also administered on the day of data collection to objectively assess each centre’s physical activity-related policies/environment [14,15,16]. The eight physical activity subscales include: Staff Behaviours, Sedentary Environment, Sedentary Opportunities, Portable Play Environment, Fixed Play Environment, Physical Activity Policies, Active Opportunities, and Physical Activity Training and Education (see Bower *et al*. [15] for a complete description of each subscale). Researchers quietly and unobtrusively used this tool to observe the childcare environment over the course of data collection. The EPAO document review consisted of examining the activity-related documents provided by each centre, and was completed following the day-long observation [16]. Both the observation and document review portions of the EPAO were used to tabulate the scores for each physical activity subscale, as well as the centres’ total physical activity EPAO score. Study protocol and material were approved by the Office of Research Ethics at the University of Western Ontario.

### 2.3. Statistical Analysis

All analyses were performed using SPSS (version 19). Accelerometry data were downloaded using Actical-specific software. The activity data were compared against Pfeiffer *et al*.’s [17] cut-points in 15 sec increments (sedentary (<50 counts per 15 sec epoch), light (≤50 ≤714 counts per 15 sec epoch), moderate (≥715 ≤ 1410 counts per 15 sec epoch), vigorous (≥1,411 counts per 15 sec epoch)), and then summed into minutes of daily activity. In line with previous research [18], participants who did not wear their accelerometer for a minimum of 240 min (*i.e.*, 4 hours (h)) were excluded from analysis. Physical activity per hour of wear time was calculated to account for the varying lengths of time participants spent in care.

The results of the eight physical activity subscales from the EPAO instrument were tallied using the scoring tool and guidelines provided by its creators [16]. The overall physical activity environment score (ranging from 0 to 20; higher score is more supportive) was calculated by averaging all eight physical activity subscale scores. Direct entry regression analyses were performed to describe the relationships between time spent in MVPA (dependent variable), total physical activity (TPA; dependent variable), and multiple independent variables (*i.e.*, physical activity subscale scores, overall EPAO score).

## 3. Results

Accelerometry wear time ranged from 246.00–561.00 min (*M* = 451.77, *SD* = 81.12), with mean operating hours for the childcare centres being 10.65 h (*SD* = 0.22). On average, participants spent 11.45 min engaged in daily MVPA (mean rate = 1.54 min/h, *SD* = 1.41), 132.60 min in TPA (mean rate = 17.42 min/h, *SD* = 6.17), and 305.37 min in sedentary activity (mean rate = 40.64 min/h, *SD* = 9.11). None of the participating centres had physical activity-related policies or staff training/education; consequently, the two corresponding subscales were deleted from the analysis. The Active Opportunities and the Sedentary Environment subscales were excluded from the model due to multicolinearity. Physical activity subscale scores and total physical activity EPAO scores for each participating centre are presented in Table 1.

**Table 1:** Physical Activity Subscale Scores and Total Physical Activity EPAO Score for Participating Childcare Centres.

PA Subscales	Centre 1	Centre 2	Centre 3	Centre 4	Centre 5	*M* (*SD*)
Active Opportunities	16.67	13.33	16.67	16.67	6.67	14.00 (4.35)
Sedentary Opportunities	13.33	6.67	6.67	6.67	13.33	9.33 (3.65)
Sedentary Environment	13.33	13.33	6.67	6.67	13.33	10.67 (3.65)
Portable Play Environment	5.71	11.43	5.71	11.43	5.71	8.00 (3.13)
Fixed Play Environment	8.75	13.75	8.75	10.00	10.00	10.25 (2.05)
Staff Behaviours	20.00	16.00	16.00	12.00	8.00	14.40 (4.56)
Physical Activity Training & Education	0.00	0.00	0.00	0.00	0.00	0.00 (0.00)
Physical Activity Policies	0.00	0.00	0.00	0.00	0.00	0.00 (0.00)
Total Physical Activity EPAO Score	9.72	9.31	7.56	7.93	7.13	8.33 (1.13)

Notes: All scores range from 0 to 20. Total Physical Activity EPAO Score was calculated by taking the mean of all physical activity subscales. PA = physical activity; *M* = mean; *SD* = standard deviation. The mean scores for each subscale are based on one day of observation at each childcare facility.

The model, composed of the Sedentary Opportunities, Portable Play Environment, Fixed Play Environment, and Staff Behaviours subscales, accounted for 49.3% of the variability in time spent in MVPA, and was statistically significant, *F*(4,26) = 8.28, *p* < 0.01. After examining unique contributions, Portable Play Environment and Fixed Play Environment were found to explain approximately 26.7% and 26.2% of the variability, respectively. This same model accounted for 14.1% of the variability in time spent in TPA, but was not statistically significant, *F*(4,26) = 2.23, *p* > 0.05. Detailed findings are presented in Table 2.

**Table 2:** Summary of Coefficients, Confidence Intervals, *t*-values, *p*-values, Partial Correlations, and *r*^2^ for the EPAO Physical Activity Subscales and MVPA and TPA.

	PA Subscale	*B*	95% CI (lower bound, upper bound)	*t*	*p*	Partial Correlations	*r*^2^
MVPA	Sedentary Opportunities	−0.09	(−0.27, 0.10)	−0.97	0.34	−0.19	0.04
	Portable Play Environment	0.42	(0.14, 0.70)	3.08	0.01 *	0.52	0.27
	Fixed Play Environment	−0.42	(−0.70,−0.14)	−3.04	0.01 *	−0.51	0.26
	Staff Behaviours	−0.07	(−0.20, 0.05)	−1.21	0.24	−0.23	0.05
TPA	Sedentary Opportunities	−0.58	(−1.63, 0.48)	−1.12	0.27	−0.22	0.05
	Portable Play Environment	0.43	(−1.15, 2.02)	0.56	0.58	0.11	0.01
	Fixed Play Environment	−0.07	(−1.69, 1.54)	−0.09	0.93	−0.02	0.00
	Staff Behaviours	−0.20	(−0.91, 0.52)	−0.57	0.58	−0.11	0.01

Notes: Model accounts for 49.3% of the variability in MVPA (*p* < 0.01) and 14.1% in TPA (*p* > 0.05); EPAO = Environment and Policy Assessment and Observation; MVPA = moderate-to-vigorous PA, TPA = total physical activity; *B* = unstandardized coefficients; CI = confidence interval; *r*^2^ = coefficient of determination, and is interpreted as the percentage of variation in physical activity explained by the subscale of interest; * = significant subscale (*p* < 0.05).

The mean overall EPAO score for all centres was 8.33 (range: 7.13–9.72; *SD* = 1.13). The overall physical activity EPAO score of the childcare centres accounted for 10.1% of the variation in time spent in MVPA, and was statistically significant, *F*(1,29) = 4.38, *p* < 0.05. For TPA, the model accounted for less than 1% of the variability, and was not statistically significant, *F*(1,29) = 0.97, *p* > 0.05.

## 4. Discussion

The first major findings of this study are the low levels of MVPA and TPA accumulated by preschoolers. Given that participants spent approximately 7.5 h in care on the day of data collection, the corresponding amount of time (11.45 min) in MVPA was minimal. This equates to a rate of 1.54 min/h, which is comparable with Temple and colleagues’ [18] MVPA finding of 1.76 min/h. The findings from the present study were slightly lower than those reported in Gunter *et al*.’s [19] and Rice and Trost’s [20] studies whose participants accumulated approximately 9.7 and 5.8 min/h of MVPA, respectively. Furthermore, participants from the current investigation also spent less time in MVPA than those in Pate and colleagues’ [6] paper, which reported a range of 4.4–10.2 min/h depending on the preschool facility and its characteristics. The variation observed between studies could be attributed to the longer monitoring time in Pate *et al*.’s study as well as the use of a different accelerometer (ActiGraph; Fort Walton Beach, FL, USA). 

In light of the newly developed Canadian Physical Activity Guidelines for the Early Years (which recommend 180 min of daily physical activity) [21,22], the level of TPA accumulated by this group (*i.e.*, 132.60 min) are concerning, given that preschoolers spend the majority of their day in childcare, leaving little time for additional physical activity accumulation. In an effort to achieve current physical activity guidelines, additional attention is required to ensure preschoolers are accumulating adequate amounts of activity while in care.

With regard to sedentary activity, participants spent an average of 40.64 min/h in sedentary pursuits. This number is comparable to those reported by Temple *et al*. [18], who noted the mean level of sedentary behaviour among their participants to be 39.80 min/h. Again, this outcome is disconcerting given this behaviour’s close link to negative health consequences [1]. Future research should focus on interventions that minimize sedentary opportunities within the childcare environment (e.g., limiting screen time, seated activities), especially because this type of behaviour is closely linked with a multitude of negative health consequences [23].

This study advances current literature by identifying specific attributes within the Canadian childcare environment that best support/hinder preschoolers’ physical activity participation. Given that all participants attended childcare centres within the same organization, it is not surprising that the significant predictors were those that were unique to each site (e.g., staff, equipment), as opposed to those shared by all centres (e.g., physical activity policies, training). Specifically, Sedentary Opportunities, Portable and Fixed Play Environment, and Staff Behaviours accounted for nearly half of the variability in time spent in MVPA. The inverse relationship between Sedentary Opportunities and time spent in MVPA is noteworthy, as an increase in sedentary opportunities would result in less MVPA. 

Similarly, an inverse relationship between the Fixed Play Environment and Staff Behaviours and MVPA was found. One possible explanation for the relationship between MVPA and fixed play equipment might be that typically, fixed play equipment (e.g., climbing structures, slides) do not allow for running/walking activities that are readily captured by accelerometers. This is in contrast to the opportunities for physical activity provided by portable equipment (e.g., bikes, balls) [7], which permits preschoolers to play with the material while moving. These results are echoed by Temple *et al*. [18] who also noted that the presence and/or use of fixed equipment in childcare may discourage the accumulation of increased activity among this particular age group. Interestingly, Gunter *et al*. [19] found a positive association between the presence of a variety of fixed play equipment and increased activity levels among their sample. With regard to staff behaviours, it is plausible that a lack of active prompts, or inactive role modeling, may discourage activity participation among these children. Contrastingly, Gubbels *et al*. [24] and Gunter *et al*. [19] did not find an inverse relationship with regards to staff behaviours and preschoolers physical activity in childcare. Given the current mixed evidence, additional research exploring the influence of staff behaviour on preschoolers’ activity levels in childcare should be of top priority.

This study is not without limitations. Firstly, this study used a small sample size. Secondly, only one day of physical activity data was collected. Nevertheless, no other study in Canada has objectively assessed the physical activity levels of preschoolers in care, in addition to highlighting salient environmental factors that influence this particular behaviour.

## 5. Conclusions

The findings of this study suggest that preschoolers in childcare engage in low levels of MVPA. Moreover, this research indicates that less fixed play equipment, additional portable equipment, and fewer sedentary opportunities may have a positive impact on preschoolers’ activity levels while in care. The importance of staff behaviour was also highlighted, and suggests that physical activity training should be a priority for childcare providers. The results of this investigation may also serve to better position public health specialists, health promoters, and early years stakeholders to modify this unique setting in an effort to support healthy growth and development of young children. Making such amendments to an environment in which preschoolers spends a large proportion of their time, will undoubtedly help set the foundation for an active childhood and adolescence. Future research should examine the activity levels of a larger, more diverse sample of preschoolers attending various childcare centres.

## References

[1] Timmons B.W., Naylor P.J., Pfeiffer K.A. (2007). Physical activity for preschool children—How much and how?. Appl. Physiol. Nutr. Metab..

[2] Tucker P. (2008). The physical activity levels of preschool-aged children: A systematic review. Early Child. Res. Q..

[3] Malina R.M. (2011). Physical activity and fitness: Pathways from childhood to adulthood. Amer. J. Hum. Biol..

[4] Cleveland G., Forer B., Hyatt D., Japel C., Krashinsky M. (2008). New evidence about child care in Canada: Use patterns, affordability and quality. IRPP Choices.

[5] Bushnik T. (2010). Child Care in Canada. Children and Youth Research Paper Series 2006 (89-599-MIE-No. 003).

[6] Pate R.R., Pfeiffer K.A., Trost S.G., Ziegler P., Dowda M. (2004). Physical activity among children attending preschools. Pediatrics.

[7] Gordon E.S., Tucker P., Burke S.M., Carron A.V. (2013). Effectiveness of physical activity interventions for preschoolers: A meta-analysis. Res. Quart. Exercise Sport.

[8] Cardon G., van Cauwenberghe E., Labarque V., Haerens L., de Bourdeaudhuij I. (2008). The contribution of preschool playground factors in explaining children’s physical activity during recess. Int. J. Behav. Nutr. Phys. Act..

[9] Dowda M., Pate R.R., Trost S.G., Almeida M.J.C., Sirard J.R. (2004). Influences of preschool policies and practices on children’s physical activity. J. Community Health.

[10] Brown W.H., Pfeiffer K.A., McIver K.L., Dowda M., Addy C.L., Pate R.R. (2009). Social and environmental factors associated with preschoolers’ nonsedentary physical activity. Child Develop..

[11] Geoffroy M.C., Power C., Touchette E., Dubois L., Boivin M., Seguin J.R., Tremblay R.E., Cote S.M. (2012). Childcare and overweight or obesity over 10 years of follow-up. J. Pediatr..

[12] Colley R., Garriguet D., Adamo K., Carson V., Janssen I., Timmons B., Tremblay M. (2013). Physical activity and sedentary behavior during the early years in Canada: A cross-sectional study. Int. J. Behav. Nutr. Phys. Act..

[13] Cliff D.P., Reilly J.J., Okely A.D. (2009). Methodological considerations in using accelerometers to assess habitual physical activity in children aged 0–5 years. J. Sci. Med. Sport.

[14] Benjamin S., Neelon B., Ball S., Bangdiwala S., Ammerman A., Ward D. (2007). Reliability and validity of a nutrition and physical activity environmental self-assessment for child care. Int. J. Behav. Nutr. Phys. Act..

[15] Bower J.K., Hales D.P., Tate D.F., Rubin D.A., Benjamin S.E., Ward D.S. (2008). The childcare environment and children’s physical activity. Amer. J. Prev. Med..

[16] Ward D., Hales D., Haverly K., Marks J., Benjamin S., Ball S., Trost S.G. (2008). An instrument to assess the obsogenic environment of child care centers. Am. J. Health Behav..

[17] Pfeiffer K.A., McIver K.L., Dowda M., Almeida M.J.C., Pate R.R. (2006). Validation and calibration of the Actical accelerometer in preschool children. Med. Sci. Sport. Exercise.

[18] Temple V.A., Naylor P.J., Rhodes R.E., Higgins J.W. (2009). Physical activity of children in family child care. Appl. Physiol. Nutr. Metab..

[19] Gunter K.B., Rice K.R., Ward D.S., Trost S.G. (2012). Factors associated with physical activity in children attending family child care homes. Prev. Med..

[20] Rice K.R., Trost S.G. (2013). Phsysical activity levels among children attending family day care. J. Nutr. Behav. Educ..

[21] Tremblay M.S., Leblanc A.G., Carson V., Choquette L., Gorber S.C., Dillman C., Duggan M., Gordon M.J., Hicks A., Janssen I. (2012). Canadian physical activity guidelines for the early years (aged 0–4 years). Appl. Physiol. Nutr. Metab..

[22] Canadian Society for Exercise Physiology (2012). Canadian Physical Activity Guidelines for the Early Years (0–4 years). http://www.csep.ca/CMFiles/Guidelines/CSEP-InfoSheets-early-years-ENG.pdf.

[23] Tremblay M.S., LeBlanc A.G., Carson V., Choquette L., Connor Gorber S., Dillman C., Duggan M., Gordon M.J., Hicks A., Janssen I. (2012). Canadian sedentary behaviour guidelines for the early years (aged 0–4 years). Appl. Physiol. Nutr. Metab..

[24] Gubbels J.S., Kremers S.P.J., van Kann D.H.H., Stafleu A., Candel M.J., Dagnelie P.C., Thijs C., de Vries N.K. (2011). Interaction between physical environment, social environment, and child characteristics in determining physical activity at child care. Health Psychol..

